# PopMortGen-India: an excel based tool for preparing population mortality files from life tables for relative survival analysis for cancer patients from India

**DOI:** 10.3332/ecancer.2026.2082

**Published:** 2026-02-24

**Authors:** Dipak K Das, Shivraj R Tandale, Atul M Budukh

**Affiliations:** 1 Division of Medical Records and Cancer Registries, Centre for Cancer Epidemiology, Advanced Centre for Treatment, Research and Education on Cancer (ACTREC), Tata Memorial Centre, Kharghar 410210, Navi Mumbai, India; 2 Homi Bhabha National Institute, Training School Complex, Anushakti Nagar 400094, Mumbai, India

**Keywords:** relative survival, PopMort, survival analysis, cancer survival, cancer epidemiology

## Abstract

Relative survival (RS) is an essential statistic for evaluating cancer outcomes utilising data from population-based cancer registries (PBCRs). Despite India having 52 PBCRs, the availability of survival information is constrained, one of the challenges being producing population mortality (PopMort) files required for RS calculations. To address this challenge, we created an Excel-based application (PopMortGen-India) that automates the generation of PopMort files from abridged life tables supplied by India’s Sample Registration System (SRS). The program calculates age- and sex-specific mortality rates by inverting traditional life table formulas and determines survival probabilities for any particular single-year age. The application, developed in Microsoft Excel, employs organised worksheets, formula-driven automation and macros to facilitate the effortless creation of PopMort files, compatible with statistical software such as STATA, R and SAS. ‘PopMortGen-India’ markedly decreases the time needed to produce PopMort files, reducing it from several hours manually to less than 10 minutes for a decade-long dataset. Utilising the life tables of rural Punjab and Maharashtra, PopMort files were generated swiftly and precisely. RS estimates obtained from these files in STATA are closely aligned with published data, including age-standardised RS statistics. The tool offers a quick, precise and scalable method for producing PopMort data vital for RS analysis. Its alignment with manual techniques, coupled with substantial time efficiency, renders it especially appropriate for implementation in resource-limited environments. We propose its formal implementation into PBCR procedures and capacity-building measures to standardise and improve survival statistics among cancer registries in India.

## Introduction

Some of the most important statistics presented for understanding a disease’s epidemiology in an area, are its incidence, mortality, prevalence and survival at a population level. Incidence, mortality and prevalence data can be collected through routine disease surveillance systems such as population-based cancer registries (PBCRs). Survival calculations, however, require long-term follow-up of the incidence cases and linkage with mortality data [1].

PBCRs provide valuable data for conducting survival studies, enabling researchers to monitor cancer care services in the population and survival trends, which will be useful in formulating policy decisions [2]. The population-based survival rate of a disease represents the average prognosis of the disease in the population. Hence, theoretically, it reflects the effectiveness of cancer care and policy in the region. It is one of the most important parameters used to evaluate disease control programmes and policies. Survival analysis also plays a crucial role in evaluating the impact of cancer control activities on the population. Studies have shown that early diagnosis significantly improves survival rates by facilitating timely and appropriate treatment [3]. Identifying key determinants of survival, such as demographic characteristics, lifestyle factors and tumour biology, enables the development of targeted interventions aimed at reducing disparities in cancer outcomes.

Survival analysis is a key statistical approach used in cancer research to estimate patient outcomes, assess treatment efficacy and compare survival trends across demographic and clinical subgroups [4]. There are several types of survival estimates, the most commonly calculated one being observed survival. It describes the proportion of individuals who survived for a defined period after their disease diagnosis (in case of population-based studies). This rate is simple to compute and is facilitated by numerous statistical packages in commonly utilised software platforms, including SPSS, STATA, SAS, R and Python. A major limitation of this rate is that it accounts for all deaths, regardless of the cause of death. The interest in conducting a population-based survival study for a disease is to describe the mortality attributed to only that particular disease. Hence, if information on the cause of death is available, calculation of a corrected survival rate is recommended.

In several low- and middle-income countries (LMICs), including India, the combination of tough terrains and constrained resources causes essential patient follow-up exceptionally challenging. Sankaranarayanan *et al* [5] also highlighted that follow-up frequently brings difficulties even in urban settings. Information on the cause of death is often unavailable. Hence, computation of a corrected survival rate is not possible for such regions.

An alternate measure that can be used in such situations is relative survival (RS). It is the ratio of observed survival to the expected survival of the study population. It assumes that if not for the disease, the survival of the study participants and the general population of the region would have been the same [1]. Thus, the relative difference in survival is due to the disease alone. RS compares the survival of cancer patients with that of the general population, adjusting for expected mortality based on demographic factors. It provides an estimate of cancer-specific survival without requiring cause-of-death information, making it a valuable tool in epidemiological studies [2, 6, 7].

RS analysis is a widely used method in population-based cancer research to estimate the survival of patients in the absence of data on causes of death. A requirement for the calculation of RS is the expected survival probabilities of the general population that can be obtained from the general population life-tables by multiplying published annual survival probabilities corresponding to age, sex and calendar year. This file is known as a Population Mortality (PopMort) file [8]. Generation of a PopMort file involves calculating and smoothening of life tables, which is a time-taking task as life table does not provide mortality rate directly. Preparing such files manually is time-consuming and error-prone, especially when data for multiple calendar years and demographic strata are needed.

Many researchers face challenges in producing the PopMort database essential to RS estimations. This difficulty is most apparent in the creation of cancer registry reports or the implementation of cancer registry enhancement initiatives, where the absence of a PopMort file often leads to the exclusion of survival statistics. To resolve this issue and enable population-based survival analysis in resource-constrained environments, we created an Excel-based application that produces PopMort files utilising abridged life tables. This research delineates the use of this instrument for computing RS.

## Materials and methods

PopMortGen-India was developed to automate the conversion of abridged life table data into PopMort file format for use in statistical software packages such as STATA, R and SAS. The tool was developed utilising Microsoft Excel, employing essential functions, structured worksheets, automation through formulaic logic and macros to enhance functionality.

PopMort file mainly requires i) rate (General PopMort rate) and ii) probability (probability of surviving of the general population), with both variables provided in a combination of year (first incidence/occurrence year to last follow-up year of the study population), age (0–100 years) and sex (male and female) [7, 8].

The mortality rate must be estimated from a life table. In India, the Sample Registration System (SRS) provides abridged life tables, which do not directly provide the mortality rate [9]. Abridged life table provides the _n_q_x_ column [9, 10]. So the mortality rate (_n_M_x_) must be calculated by reversing the formula of calculation of _n_q_x_, as mentioned in equations 1 and 2 [10].


qxn=n·Mxn1+fxn·Mxn(1)

Mxn=qxnn−fxn·qxn(2)

where *_n_q_x_* is the probability that a person of exact age ‘*x*’ will die within ‘*n*’ years; *n* is the age of person; *_n_M_x_* the central death rate of a population that is between exact ages *x* and *x* + *n*; and *_n_f_x_* is the separation factor which is 1/2 for 0–4 age group and 5/2 for each age group of 5–94 age group [10].

As the mortality rate and probability of surviving are mathematically related, we only need one of them. Using the mortality rate of a particular age group, we can easily calculate the probability of survival of that group using the term ‘Probability = Exp(-Rate)’ [8].

**Figure 1.**
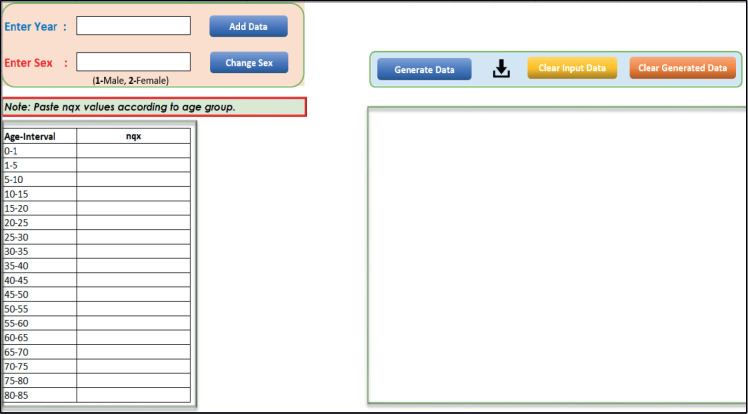
Interface snapshot of the PopMortGen-India.

As the abridged life table provides an age-group-wise life table, our calculated rate and probability will also represent that age group. Therefore, we need to smoothen them for a single year. Here we have taken the same results for all the ages in that group as suggested by Dickman and Coviello [8]. For example, if we calculated the mortality rate and probability for the age group of 15–20, the same mortality rate and probability will be continued for each year of 15–20 age group. As the life table presented by SRS is in the range of year, assuming that it will best represent of midyear and we take this life table for midyear. Thus, the life table of 2015–2019 will be the best representation of 2017, likewise, the life table of 2016–2020 will be the best representation of 2018. If data is not available for recent years, it is standard practice to assume the probabilities are the same as those most recently available [8]. The latest life table produced by SRS is for the period 2016–2020, so the same data shall be continued for 2018 and onwards [9]. Figure 1 illustrates the user interface of PopMortGen-India, alongside Table 1 gives a comprehensive description of each tab and function inside the tool, highlighting their specific roles in the process.

### Steps for preparing the PopMort file

- Open this PopMortGen-India and go to the ‘Input’ sheet. In the 'Enter Year' field, type the starting year (first incidence/diagnosis year) and the ending year (last follow-up year).
- Specify the sex based on the life table data. Enter 1 for male and 2 for female. You can also use the ‘Change Sex’ button to toggle between male and female.
- Open the relevant life table according to your requirements. For example, if you're working on rural Maharashtra data, use the life table specific to rural Maharashtra.
- Copy the ‘nqx’ values (probability of dying between ages) for all age groups from the selected life table based on the selected sex and paste them into the ‘nqx’ column in the tool.
- Click the ‘Add Data’ button to input the data. The entered information will appear in the box on the right side of the same sheet.
- Switch to the opposite sex using the ‘Change Sex’ button and repeat the process: copy and paste the appropriate ‘nqx’ values for all age groups and click ‘Add Data’ again to input the second set of data.
- For each additional year, return to the ‘Enter Year’ tab, input the new year and repeat steps 2–6. Ensure all required years are added properly using the ‘Add Data’ button.
- Once all data has been entered, click on the ‘Generate Data’ button. The generated PopMort data will be displayed in a new sheet named ‘Output_data.’
- ix. To save the generated data, click on the ‘ 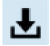
’ (download) icon on the ‘Input sheet’. A popup window will appear asking if you want to save the file. Click ‘Yes’, choose your desired location and save the file with a preferred name (typically ‘PopMort’).

The rural Punjab abridged life table (which came from SRS) was utilised to create a PopMort file for the period 2009–2024 using this Excel-based solution, with the full procedure accomplished in less than 10 minutes. Follow-up data on prostate cancer cases from the Sangrur and Mansa districts in rural Punjab were acquired from the study conducted by Budukh *et al* [11]. RS was subsequently computed utilising the created PopMort file, and the analysis was performed using STATA software. Furthermore, PopMort files for rural Maharashtra were generated utilising the relevant rural Maharashtra life table. These files were sent to two postgraduate students to assess the comparative survival rates of oral and breast cancer in Ratnagiri, Maharashtra, as part of their dissertation research.

## Results

**Figure 2.**
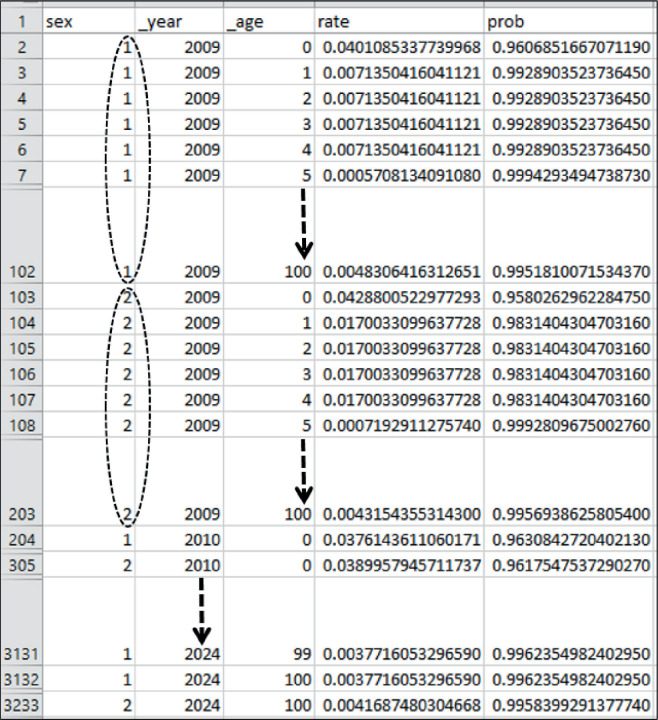
Overall output layout of the PopMort file created by PopMortGen-India. Footnote: The PopMort file includes data for ages 0–100, covering both sexes, from the first incidence year (2009) through the last follow-up year (2024).

PopMortGen-India facilitated the efficient generation of PopMort files from life tables for specified study regions, markedly decreasing the file preparation time from several hours manually to under 10 minutes for a 10-year dataset. Figure 2 exhibits the comprehensive output structure of the generated PopMort file, highlighting the final layout and structure of the variables.

The PopMort file has been completed specifically for the designated area of research. This file is now eligible for conversion into an appropriate format and further importation into statistical software such as STATA, SAS or R for the execution of RS analysis.

Table 2 displays the published RS Rates (RSRs) and estimated RSR, accompanied by 95% confidence intervals (CI), at 1, 3 and 5 years across various age groups. For all age cohorts (45–64, 65–74 and ≥75 years), the projected RSR practically match with the reported rates, and the published statistics remain within the respective 95% CI of the estimates. In the total population (*n* = 171), the reported RSR were 69.4 at 1 year, 39.8 at 3 years and 30.3 at 5 years. The calculated RSR were 69.41 (95% CI: 61.41, 76.08) at 1 year, 39.77 (95% CI: 31.68, 47.72) and 30.01 (95% CI: 21.98, 38.44) at 5 years.

Table 3 illustrates the published and estimated age-standardised overall RS (ASRS) rates at 1, 3 and 5 years for all age groups and persons aged 0–74 years. The projected ASRS values align strongly with the reported rates across all time points. The published ASRS for the overall population was 69.4%, 39.8% and 30.3% at 1, 3 and 5 years, respectively, whereas the calculated ASRS was 69.43% (95% CI: 61.44–76.10), 39.75% (95% CI: 31.63–47.74) and 30.22% (95% CI: 22.21–38.62). Correspondingly, for the 0–74 age groupings, the reported ASRS was 72.4%, 40.7% and 31.6%, which matches closely with the estimated values of 72.39% (95% CI: 64.63–78.73), 40.81% (95% CI: 32.85–48.60) and 31.71% (95% CI: 23.97–39.71).

Our generated PopMort files for rural Maharashtra were utilised by two postgraduate students who previously calculated RS using a manually created PopMort file for their dissertations on oral and breast cancer survival in Ratnagiri and Sindhudurg, Maharashtra. The outcomes determined from the new PopMort dataset matched with the manually calculated estimations. Both dissertations were submitted to the Homi Bhabha National Institute in India. PopMortGen-India has also been utilised by researchers at the Centre for Cancer Epidemiology, Tata Memorial Centre, India, to produce PopMort files for Muzaffarpur (Bihar) and checked with Sangrur (Punjab) data. Their population-based RS has been documented in their respective reports [12, 13]. Table 4 delineates the comparison between manual approaches and the PopMortGen-India for generating PopMort files, focusing on time efficiency, data processing, error rates, consistency and additional parameters.

## Discussion

RS is an important indicator of the availability and accessibility of diagnostic and treatment facilities in a population, besides simple disease prognosis. PBCRs in resource-constrained regions face methodological difficulties in calculating RS due to the tedious work involved in the generation of the PopMort file. Through this PopMortGen-India, we aim to enhance the efficiency of data analysis and enable faster reporting of population-based disease survival outcomes. The tool provides a significant benefit in time efficiency, requiring under 10 minutes to generate a 10-year PopMort file, a process that often requires 2–5 hours when performed manually. The tool was validated via various case applications, encompassing a rural Punjab life table for prostate cancer survival analysis in Sangrur and Mansa districts [11], as well as a rural Maharashtra life table for oral and breast cancer survival analysis in Ratnagiri and Sindhudurg districts, Maharashtra.

The computed RS estimates obtained from the PopMortGen-India closely matched with published values across several age groups and subsequent research lengths. The prostate cancer dataset from Punjab [11] indicated that both age-specific and ASRS exhibited negligible variation from the stated rates, with all reported values residing within the 95% CI of the estimations. Likewise, findings from two master's dissertations, which initially depended on manually constructed PopMort files, aligned with those produced by the automated process. These findings collectively validate the precision and reliability of PopMortGen-India in generating dependable PopMort data for survival analysis.

The PopMortGen-India has shown practical utility in real-world research environments, such as cancer registries and academic institutions, including researchers at the Centre for Cancer Epidemiology, Tata Memorial Centre. Despite the existence of 52 PBCRs in India [14], very few survival statistics. This constraint stems from issues including difficulty in follow up completion, deficiency of specialists, restricted resources and the shortage of accessible analytical tools for survival estimation. Comparable difficulties have also been documented in other LMICs [5, 15]. To reduce this deficiency, this present tool enables registries to effortlessly produce PopMort files utilising life tables. This PopMortGen-India tool is particularly beneficial in limited-resource settings and can enhance the accessibility of survival insights across registries. Collaboration with IARC and its regional partners in Mumbai for training can enhance registry capability for survival analysis.

The PopMortGen-India, while efficient and user-friendly, has some limitations. It relies on the availability and quality of life tables, which in India are published with significant delays by the SRS. Notably, no updated life tables have been released after the COVID-19 pandemic, so recent mortality shifts, including COVID-19-related deaths, are not captured. The tool also assumes stable mortality trends and cannot adjust for sudden changes. Currently, it is limited to Indian populations; future versions will be developed to support life tables and mortality data from other countries.

In conclusion, this ‘PopMortGen-India’ tool provides an efficient, dependable and scalable solution for the preparation of PopMort data for application in RS studies. Its alignment with manual calculations, coupled with substantial time efficiency and user-friendliness, renders it an invaluable tool for cancer epidemiologists, public health researchers and registry data analysts, especially in low-resource or high-burden environments where prompt and precise survival analysis is important.

### Tool (PopMortGen-India) availability

The tool (PopMortGen-India) developed in this study is available upon reasonable request by contacting the corresponding author via email. Following confirmation of publication, the PopMortGen-India will also be made publicly accessible through the Centre for Cancer Epidemiology, Tata Memorial Centre website and the download link will be shared accordingly. The user can get detailed information about generation of PopMort data through YouTube https://youtu.be/00Eo8m7dRag

**List of abbreviations**

ASRSR: Age-Standardised Relative Survival Rates, CI: Confidence Intervals, LMICs: Low- and Middle-Income Countries, PBCRs: Population-Based Cancer Registries, PopMort: Population Mortality, RS: Relative survival, RSR: Relative Survival Rates, SRS: Sample Registration System.

## Conflicts of interest

None declared.

## Funding

None declared.

## Author contributions

Dipak K Das: Conceptualisation, Methodology, Software, Formal analysis, Validation, Writing - Original draft.

Shivraj R Tandale: Conceptualisation, Software.

Atul M Budukh: Supervision, Conceptualisation, Writing - Review & editing.

## Ethical approval

Not applicable.

## Novelty and impact

The study introduces an Excel-based tool (PopMortGen-India) that automates the generation of PopMort files based on India’s life tables for cancer RS. It significantly minimises time, errors and inconsistencies, facilitating precise, standardised outputs in <10 minutes. The tool improves cancer surveillance capabilities in limited resource environments, facilitating accurate public health decisions and formulation of policies.

## Figures and Tables

**Table 1.** Function and description of tabs/fields in the PopMortGen-India.

Sl.no.	Tab/Sign	Description
1	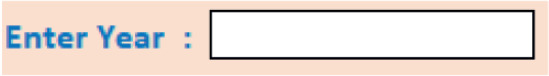	This is the field for entering the required year. For example, to generate a PopMort file for 2015, type ‘2015’ in this box.
2	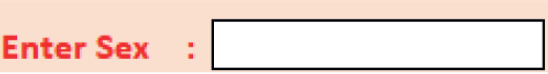	Enter the sex code based on the life table: 1 for male and 2 for female.
3	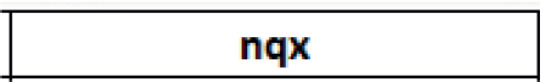	Input the _n_q_x_ values (the probability that a person aged exactly *x* will die within *n* years) from the appropriate life table. Ensure the values match the selected sex and include all age groups.
4	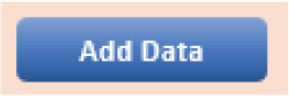	Click this tab to add the entered inputs. The data will be displayed in the ‘Input Data’ box on the same sheet.
5	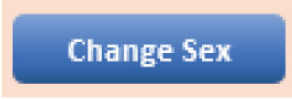	This button is used to switch between male and female. When clicked, the sex will toggle and the _n_q_x_ input field will automatically reset.
6	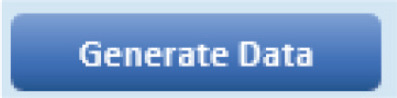	Use this tab after all entries have been added to generate the PopMort file. The output will appear in a new sheet named ‘output_data.’
7	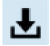	Click this to save the generated data to a separate file. A prompt will appear to confirm saving and specify the file location and name.
8	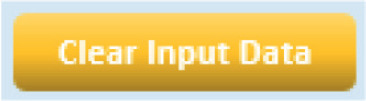	This tab clears all previously inputted data from the Input sheet.
9	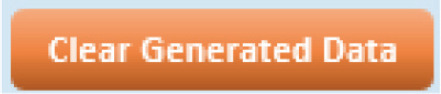	This tab clears the generated output data.
10	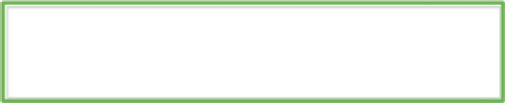	This section displays all added _n_q_x_ values categorised by year, sex and age group.

**Table 2.** Published and estimated relative-survival-rates (RSR) for prostate cancer diagnosed in Punjab, India (2013–2016).

Variable (*n*)	Published RSR (11)			Estimated RSR (95%CI)		
	1 year	3 year	5 year	1 year	3 year	5 year
Age (year)						
45–64 (64)	70.1	38.2	30.6	70.13 (56.92, 79.98)	38.22 (25.91, 50.40)	30.72 (19.10, 43.10)
65–74 (54)	75.1	43.7	32.7	75.07 (60.08, 85.09)	43.64 (28.94, 57.44)	32.36 (18.30, 47.26)
* ≥*75 (53)	62.8	37.7	27.4	62.77 (47.03, 75.02)	37.68 (22.84, 52.45)	25.96 (11.71, 42.83)
*Total (171)*	69.4	39.8	30.3	69.41 (61.41, 76.08)	39.77 (31.68, 47.72)	30.01 (21.98, 38.44)

**Table 3.** Published and estimated age-standardised overall-relative-survival-rates (ASORSR) for prostate cancer diagnosed in Punjab, India (2013–2016).

Age (year)	Published ASORSR (11)			Estimated ASORSR (95%CI)		
	1 year	3 year	5 year	1 year	3 year	5 year
All ages	69.4	39.8	30.3	69.43 (61.44, 76.10	39.75 (31.63, 47.74)	30.22 (22.21, 38.62)
0–74 years	72.4	40.7	31.6	72.39 (64.63, 78.73)	40.81 (32.85, 48.60)	31.71 (23.97, 39.71)

**Table 4.** Comparison of manual methods and PopMortGen-India tool for creating PopMort files.

Aspect	Manual method	PopMortGen-India
Time required	Requires several hours (2–5 hours) to create a 10-year file	Takes approximately 10 minutes to generate a 10-year PopMort file
Data processing	Involves manual merging and formatting in Excel	Fully automated processing and file generation
Error risk	High risk of manual entry and calculation errors	Minimises error risk through automated computations
Data consistency	Greater chance of inconsistencies across variables and files	Ensures high consistency through standardised processes
Analysis timeline	Delays data analysis due to time-consuming file preparation	Enables faster analysis with quick file creation
Technical skill requirement	Requires advanced Excel handling and attention to detail	Can be used by researchers with basic Excel knowledge
Scalability	Difficult to scale for large datasets or multiple regions	Easily scalable for multiple years and regions
Adaptability	Time-consuming to update if new life tables are introduced	Easy to update with new life tables by modifying input sheet
Reproducibility	Difficult to reproduce exactly due to manual steps	High reproducibility due to automated and standardised process
